# Combined influence of nonalcoholic fatty liver and body size phenotypes on diabetes risk

**DOI:** 10.1186/s12933-015-0306-0

**Published:** 2015-10-29

**Authors:** Tingting Du, Xuefeng Yu, Gang Yuan, Jianhua Zhang, Xingxing Sun

**Affiliations:** Department of Endocrinology, Tongji Medical College, Tongji Hospital, Huazhong University of Science and Technology, 430030 Wuhan, China; Department of Anesthesiology, Tongji Medical College, Tongji Hospital, Huazhong University of Science and Technology, 430030 Wuhan, China

**Keywords:** Nonalcoholic fatty liver disease, Overweight, Obesity, Diabetes

## Abstract

**Background:**

We aimed to determine the association between nonalcoholic fatty liver disease (NAFLD) and diabetes risk among body size phenotypes which was based on cross-classification of body mass index (BMI) categories (normal or overweight/obesity) and metabolic status (metabolically health or metabolically at-risk).

**Methods:**

We conducted a cross-sectional analysis using a cohort of 10,761 apparently healthy Chinese adults who underwent comprehensive health checkups including abdominal ultrasonography. Subjects were classified as metabolically at-risk by having any two of the following, consistent with the Adult Treatment Panel-III metabolic syndrome definition: (1) systolic/diastolic blood pressure ≥130/85 mmHg, (2) triglycerides ≥1.7 mmol/L, (3) fasting blood glucose ≥5.6 mmol/L, (4) HDL-cholesterol ≥1.0/1.3 mmol/L for men/women.

**Results:**

Among participants without metabolically at-risk, multivariate-adjusted odds ratios (ORs) for diabetes from NAFLD compared with those without NAFLD in the normal-weight (BMI <23 kg/m^2^) and overweight/obese (BMI ≥23 kg/m^2^) group were 2.10 (1.85–3.93) and 1.85 (1.35–2.53), respectively. Among participants with metabolically at-risk, the significant association between NAFLD and diabetes was lost, regardless of obesity status. There were only 27.1 % subjects with the presence of the three factors (overweight/obesity, NAFLD, and metabolically at-risk) occurring together, while the three factors occurring together was common (56.16 %) in diabetic individuals. The multivariate-adjusted ORs for diabetes were 1.1 (0.61–1.98) for overweight/obesity, 2.23 (1.05–5.14) for NAFLD, and 8.04 (5.0–12.09) for metabolically at-risk. The OR for the presence of all the three factors occurring together was 23.22 (13.96–38.63).

**Conclusions:**

NAFLD was associated with diabetes risk among participants without metabolically at-risk. The clustering of overweight/obesity, NAFLD, and metabolically at-risk is common in diabetic subjects and strikingly and markedly increases the diabetes risk.

## Background

Along with the increasing westernization of diet, physical inactivity, and the obesity epidemic [1], the worldwide prevalence of nonalcoholic fatty liver disease (NAFLD) is increasing rapidly, affecting between 15 and 30 % of adults [2, 3]. NAFLD is characterized by significant lipid deposits in the liver in patients with absence of excessive alcohol consumption. It has been considered as the hepatic manifestation of metabolic syndrome. Although obesity has contributed substantially to the burden of NAFLD, approximately 15 % of non-obese individuals may be encountered with NAFLD [4]. Clinic significance of NAFLD has been evaluated in non-obese patients [5–7]. Some data show that NAFLD is independently associated with insulin resistance [5, 6] and metabolic disorders [7] in normal-weight individuals. A recent study suggests that the associations between NAFLD and metabolic disorders differ between non-obese and obese patients, with the associations stronger in non-obese than in obese individuals [8]. On the other hand, components of metabolic syndrome (such as hyperglycemia, dyslipidemia, or hypertension) are intimately related to the development of NAFLD [9, 10]. However, NAFLD is not a rare disease in persons without such risk factors [9]. Evidence shows that NAFLD is only associated with increased arterial stiffness [11] or carotid intima-media thickness [12] in the presence of metabolic risk factors. However, data also demonstrate that NAFLD forecasts an increased risk of cardiovascular disease (CVD) [13] independent of metabolic syndrome. Taken together, the clinic significance of NAFLD may vary both by the obesity status and metabolic status. Body mass index (BMI) is a poor indicator of body fat distribution, as evidenced by the occurrence of the variation in the burden of metabolic disorders, diabetes, and CVD among individuals with similar BMI [14]. The concomitant presence of body size and metabolic status (that is, body size phenotype) can provide information on the distribution of body fat [15]. Excess visceral or ectopic fat accumulation can modulate cardiometabolic risk due to their greater endocrine activity [16, 17]. Emerging evidence show that visceral or ectopic fat accumulation is a strong correlate of NAFLD [18]. Until now, few studies have examined CVD risk factors and diabetes risk associated with NAFLD among body size phenotypes.

Hence, we aim to evaluate the association of NAFLD with CVD risk factors and diabetes risk among body size phenotypes.

## Methods

### Study population

The study participants were Chinese employees and retired workers aged 20–100 years from the Wuhan Iron and Steel Company (WISCO), which is one of the largest iron and steel companies in China. In WISCO, the Industrial Safety and Health Law requires employees and retired workers to receive periodic health evaluations at the WISCO General Hospital (Wuhan, China). The present cohort included all employees and retired workers who received a comprehensive health examination (including abdominal ultrasonography) at the Healthcare system, WISCO general Hospital, between June 2008 and December 2010 (n = 15,753).

All subjects were asked to complete a standard questionnaire that gathered information on age, sex, cigarette smoking and alcohol consumption habits, histories of current and previous illness, and medical treatment. We excluded 4992 participants from this study, comprising 1493 participants who were taking medications for hypertension, diabetes, dyslipidemia, or hyperuricemia, 1271 with alcohol consumption in amounts >70 g/week for women (73) and >140 g/week for men (1198), 857 participants with hepatitis B surface antigen (HBsAg) positivity, and 1758 missing information on age, sex, anthropometric assessment, components of metabolic syndrome, test results for HBsAg, or liver ultrasound scans. As some individuals met more than one exclusion criteria, the remaining available 10,761 participants (6901 men and 3860 women) were included in our data analysis. The fact that men accounted for 64.1 % of total participants was in consistent with the proportion of male employees at WISCO. According to the Private Information Protection Law, information that might identify subjects was safeguarded by the Health Examination Center. This study was approved by the institutional review board of WISCO general Hospital. Because we only retrospectively accessed a de-identified database for purposes of analysis, informed consent requirement was exempted by the institutional review board.

### Anthropometric and biochemical measurements

Anthropometric measurements, including weight, height, and blood pressure (BP) were measured following standardized protocols from the World Health Organization (WHO). Weight was measured with the participants wearing light clothing and height was measured without shoes. BMI was calculated as weight (in kilograms) divided by the square of height (in meters). According to the WHO criteria for Asians [19], subjects were classified as normal weight (BMI of 18.5–22.9 kg/m^2^), and overweight/obesity (BMI ≥23 kg/m^2^). Participants’ seated BP was measured twice for every 5 min on the right arm after 5 min of rest by trained nurses with a sphygmomanometer. The mean of the two readings was used in data analysis.

Overnight fasting (at least 8 h) blood samples were collected from the antecubital vein of each individual. Biochemical measurements, including assessment of fasting blood glucose (FBG), total cholesterol (TC), triglycerides (TG), low-density lipoprotein cholesterol (LDL-C), high-density lipoprotein cholesterol (HDL-C), alanine aminotransferase (ALT), and hepatitis viral antigen/antibody, were measured enzymatically on an autoanalyzer (Hitachi 7600, Ltd., Tokyo, Japan). All the blood measurements were followed the same protocol.

### Assessment of NAFLD

Ultrasound tests were performed by trained sonographers using a high-resolution, real-time scanner (model SSD-2000; Aloka Co., Ltd., Tokyo Japan). One experienced radiologists used standard criteria in evaluating the images for the presence or absence of hepatic fat [20]. Generally, the diagnosis of fatty liver was based on the presence of stronger echoes in the hepatic parenchyma compared with echoes in the kidney or spleen parenchyma [21].

### Assessment of metabolic health status

Subjects were classified as metabolically at-risk by having any two of the following, consistent with the Adult Treatment Panel-III (ATP III) metabolic syndrome definition [22] and with previous studies [23]: (1) High BP: systolic/diastolic BP ≥130/85 mmHg, (2) High TG: TG ≥1.7 mmol/L (150 mg/dL), (3) High FBG: FBG ≥5.6 mmol/L (100 mg/dL), (4) Low HDL-C: HDL-C ≥1.0/1.3 mmol/L (40/50 mg/dL) for men/women. Waist circumference was not included in the definition because of collinearity with BMI. Subjects were classified as metabolically healthy by having zero or one risk factor. Participants were then categorized into four mutually body size phenotypes based on combinations of BMI categories and metabolic status: metabolically healthy and normal weight (MHNW), metabolically at-risk and normal weight (MANW), metabolically healthy and overweight or obese (MHOW), metabolically at-risk and overweight or obese (MAO).

According to the 2014 American Diabetes Association criteria [24], diabetes is defined as having FBG ≥7.0 mmol/L.

### Statistical analysis

All statistical analyses were conducted using SPSS software (version 12.0 for windows; SPSS, Chicago, IL, USA). Variables were presented as means and standard errors (SE) for continuous variables and as percentages for categorical variables. Based on cross-classification of four body size phenotypes and the NAFLD status, participants were categorized into eight mutually exclusive groups (MHNW with or without NAFLD, MANW with or without NAFLD, MHO with or without NAFLD, MAO with or without NAFLD). Differences in characteristics among these groups were verified by analysis of covariance or Cochran–Mantel–Haenszel Chi square statistics as appropriate. A post hoc (Scheffé method) multiple comparison was used to establish the differences between the groups, when necessary. Furthermore, a logistic regression model was used to estimate crude and adjusted odds ratios (OR) and corresponding 95 % confidence intervals (CI) for the presence of NAFLD compared with the absence of NAFLD within each body size phenotype. The three models were as follows: Model 1 was an unadjusted model. Model 2 was adjusted for age, and sex. Model 3 was additionally adjusted for TG/HDL-C (which was a good indicator of insulin resistance).

Venn diagram was constructed as a visual display of NAFLD and overweight/obesity based on BMI ≥23 kg/m^2^. Similar processes were repeated for NAFLD and metabolic at-risk.

A logistic regression model was used to examine the joint associations of body size phenotypes and the NAFLD status with diabetes risk to determine whether the associations of body size phenotypes with diabetes differ based on the presence of NAFLD. Significance was accepted at a two-tailed P < 0.05.

## Results

The prevalence of NAFLD in the MHNW, MHO, MANW, and MAO phenotype was 8.79, 31.72, 50.98, and 76.19 %, respectively.

Table 1 described clinical characteristics of the study population stratified by body size phenotypes and NAFLD status. In each body size phenotype, subjects with NAFLD showed more atherogenic lipid profile as indicated by higher levels of TG, LDL-C, TG/HDL-C, and lower levels of HDL-C, and higher FBG levels than subjects without NAFLD (all P < 0.0001); Concentrations of atherogenic lipids and FBG were significantly higher in the MHNW/with NAFLD group (that is, participants with NAFLD alone) compared with the MHO/without NAFLD group (that is, participants with overweight/obesity alone), implying that the associations of NAFLD with unfavorable lipid and glucose profiles were stronger than those of overweight/obesity. On the other hand, MHNW/with NAFLD group (that is, participants with NAFLD alone) had lower concentrations of atherogenic lipids than the MANW/without NAFLD group (that is, participants with metabolically at-risk alone), indicating that the association of NAFLD with unfavorable lipid profile was weaker than that of metabolically at-risk. For a given BMI status, the most favorable lipid profile and glucose levels were found in individuals with metabolically health/without NAFLD, and the worst atherogenic lipid profile and glucose levels were found in individuals with metabolically at-risk/with NAFLD: indicating that NAFLD and metabolically at-risk have synergistic effects on atherogenic lipid and glucose concentrations. For a given metabolic status, the differences in TG, TG/HDL-C, HDL-C, and FBG between individuals with NAFLD and without NAFLD were more striking among normal-weight subjects than among overweight/obese subjects, suggesting that NAFLD may reflect a more unfavorable distribution of body fat in normal-weight group.

**Table 1:** Adjusted means of anthropometric and biochemical variables between groups with NAFLD and without NAFLD, stratified by overweight/obesity and metabolic status

	BMI <23 kg/m^2^				BMI ≥23 kg/m^2^			
	Metabolically healthy		Metabolically at-risk		Metabolically health		Metabolically at-risk	
	Without NAFLD	With NAFLD	Without NAFLD	With NAFLD	Without NAFLD	With NAFLD	Without NAFLD	With NAFLD
N	3625	332	437	203	1776	1847	605	1936
Age (years)*	44.81 ± 0.25	48.77 ± 0.70	56.17 ± 0.69	56.41 ± 0.92	50.06 ± 0.34	50.40 ± 0.32	56.14 ± 0.59	53.61 ± 0.30
BMI (kg/m^2^)	20.59 ± 0.03	21.78 ± 0.06	21.26 ± 0.06	21.93 ± 0.07	24.75 ± 0.04	26.08 ± 0.05	25.10 ± 0.07	26.73 ± 0.06
SBP (mmHg)	116.12 ± 0.23	116.34 ± 0.75	128.48 ± 0.66	128.30 ± 0.96	120.67 ± 0.33	122.16 ± 0.33	130.23 ± 0.56	131.37 ± 0.32
DBP (mmHg)	72.90 ± 0.16	74.43 ± 0.53	81.30 ± 0.47	81.28 ± 0.68	76.30 ± 0.23	78.00 ± 0.23	82.56 ± 0.40	84.43 ± 0.23
ALT (U/L)	19.08 ± 0.23	26.58 ± 1.54	21.54 ± 0.88	25.14 ± 0.98	25.25 ± 0.83	30.32 ± 0.50	23.93 ± 0.60	35.19 ± 0.58
Total cholesterol (mmol/L)	4.40 ± 0.01	4.72 ± 0.05	4.58 ± 0.04	4.81 ± 0.06	4.58 ± 0.02	4.81 ± 0.02	4.74 ± 0.04	5.00 ± 0.02
Triglycerides (mmol/L)	0.86 ± 0.02	1.29 ± 0.07	1.78 ± 0.06	2.70 ± 0.09	1.04 ± 0.03	1.32 ± 0.03	1.96 ± 0.05	2.73 ± 0.03
LDL cholesterol (mmol/L)	2.64 ± 0.01	2.93 ± 0.04	2.73 ± 0.04	2.77 ± 0.05	2.85 ± 0.02	3.07 ± 0.02	2.84 ± 0.03	2.92 ± 0.02
HDL cholesterol (mmol/L)	1.54 ± 0.00	1.45 ± 0.02	1.31 ± 0.01	1.21 ± 0.02	1.43 ± 0.01	1.38 ± 0.01	1.23 ± 0.01	1.20 ± 0.01
Triglycerides/HDL-C	0.58 ± 0.04	0.95 ± 0.12	1.55 ± 0.10	2.47 ± 0.15	0.76 ± 0.05	1.00 ± 0.05	1.86 ± 0.09	2.66 ± 0.05
FPG (mmol/L)	4.94 ± 0.02	5.58 ± 0.07	6.04 ± 0.06	6.45 ± 0.08	5.00 ± 0.03	5.53 ± 0.03	5.67 ± 0.05	5.98 ± 0.03
Uric acid (mmol/L)	273.14 ± 1.11	294.62 ± 3.61	289.96 ± 3.15	309.97 ± 4.61	291.76 ± 1.57	314.72 ± 1.56	303.25 ± 2.69	332.81 ± 1.53

Metabolically healthy and metabolically at-risk were defined in the definition section

Data are presented as mean (SE)

Data adjusted for age and sex

*NAFLD* nonalcoholic fatty liver disease, *BMI* body mass index, *SBP* systolic blood pressure, *DBP* diastolic blood pressure, *ALT* alanine aminotransferase, *LDL* low-density lipoprotein, *HDL* high-density lipoprotein, *FPG* fasting plasma glucose

* Data was unadjusted

**Fig. 1:**
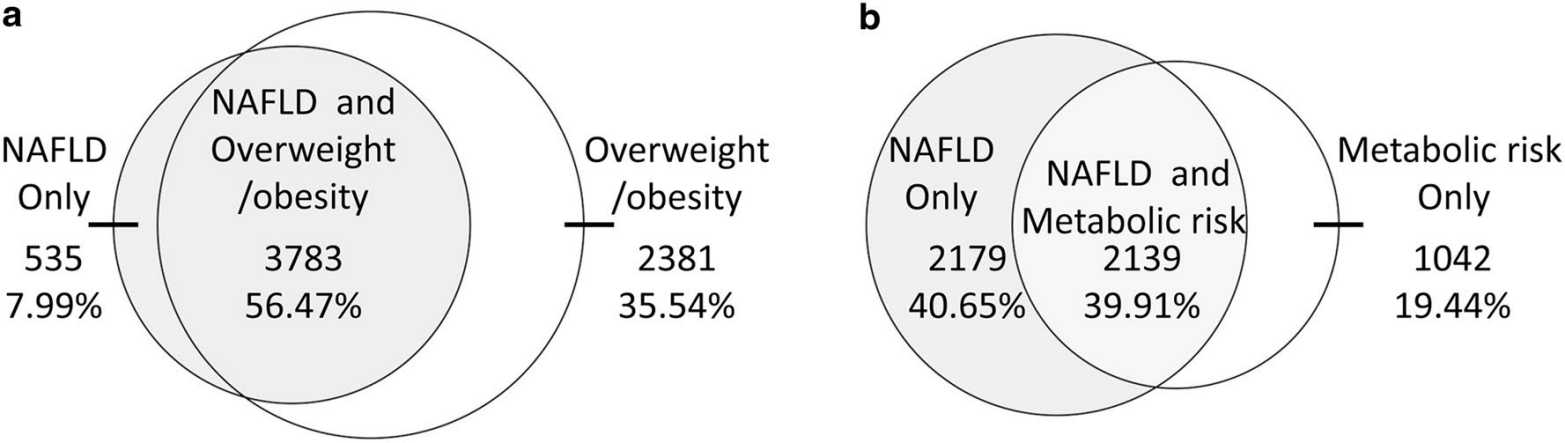
Venn Diagrams for concordance between obesity and nonalcoholic fatty liver disease (NAFLD) or between metabolically at-risk and NAFLD. Venn Diagram for a visual display of NAFLD and overweight/obesity based on BMI ≥23 kg/m^2^ in the whole population (**a**); Venn Diagram for a visual display of NAFLD and metabolically at-risk in the whole population (**b**). Metabolic risk indicates metabolically at-risk. Metabolically at-risk was defined in the Definition section

We then assessed overlap between NAFLD and overweight/obesity diagnosed by BMI ≥23 kg/m^2^. Among those subjects (6699) with either NAFLD or overweight/obesity, considerable participants (35.54 %) had overweight/obesity alone compared with 7.99 % with NAFLD alone. Moderate magnitude of overlap existed between NAFLD and overweight/obesity (56.47 %) (Fig. 1a). When analysis were stratified by the metabolic status, we found that the magnitude of overlap existed between NAFLD and overweight/obesity was higher in metabolically at-risk subgroup (70.6 %) than that in metabolically healthy group (46.7 %) (The figure was not shown).

Similarly, we further assessed overlap between NAFLD and metabolically at-risk. Low magnitude of overlap existed between NAFLD and metabolically at-risk (39.91 %) (Fig. 1b). When analysis were stratified by the BMI categories, we found that the magnitude of overlap existed between NAFLD and metabolically at-risk was higher in overweight/obese subgroup (44.12 %) than that in normal-weight group (20.88 %) (The figure was not shown).

**Fig. 2:**
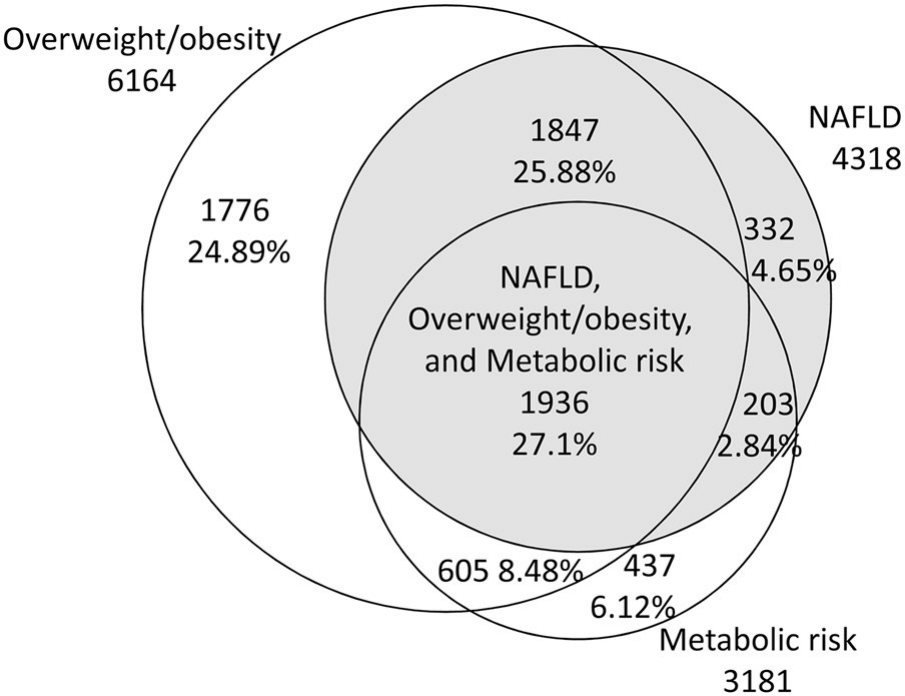
Venn diagram for a visual display of how the three factors (overweight/obesity, NAFLD, and metabolically at-risk) cluster together. Metabolic risk indicates metabolically at-risk. Overweight/obesity, NAFLD, and metabolically at-risk were defined in the definition Section

Figure 2 illustrated how the three factors of interest (NAFLD, overweight/obesity, and metabolically at-risk) cluster together. Of the 7136 subjects who were identified to have any of the three factors, there were 1936 (27.1 %) individuals with the simultaneous presence of the three factors, which occupied the largest proportion. The three factors occurred together in 56.16 % of diabetic individuals.

The ORs for diabetes risk from NAFLD compared with those without NAFLD were shown in Table 2. NAFLD was associated with diabetes in the MHNW and MHO phenotype, but the significance was lost in the MANW and MAO phenotype. ORs for diabetes from NAFLD compared with those without NAFLD in the MHNW and MHO phenotype were 2.10 (1.85–3.93) and 1.85 (1.35–2.53) (model 2), respectively. These associations persisted after additional adjustment for TG/HDL-C, a good indicator of insulin resistance (model 3).

**Table 2:** Odds ratios for NAFLD-related risk of diabetes within each body size phenotype

	Metabolically healthy normal weight	Metabolically healthy overweight or obese	Metabolically at-risk normal weight	Metabolically at-risk overweight or obese
Model 1	2.77 (1.20–6.38)	1.74 (1.28–2.36)	1.52 (0.96–2.31)	1.48 (0.82–2.64)
Model 2	2.10 (1.85–3.93)	1.85 (1.35–2.53)	1.38 (0.89–2.14)	1.41 (0.78–2.54)
Model 3	2.58 (1.97–4.24)	1.83 (1.34–2.50)	1.38 (0.87–2.17)	1.61 (0.88–2.95)

Model 1 was an unadjusted model

Model 2 was adjusted for age, and sex

Model 3 was adjusted for all variables in model 2 plus triglycerides/HDL cholesterol

**Fig. 3:**
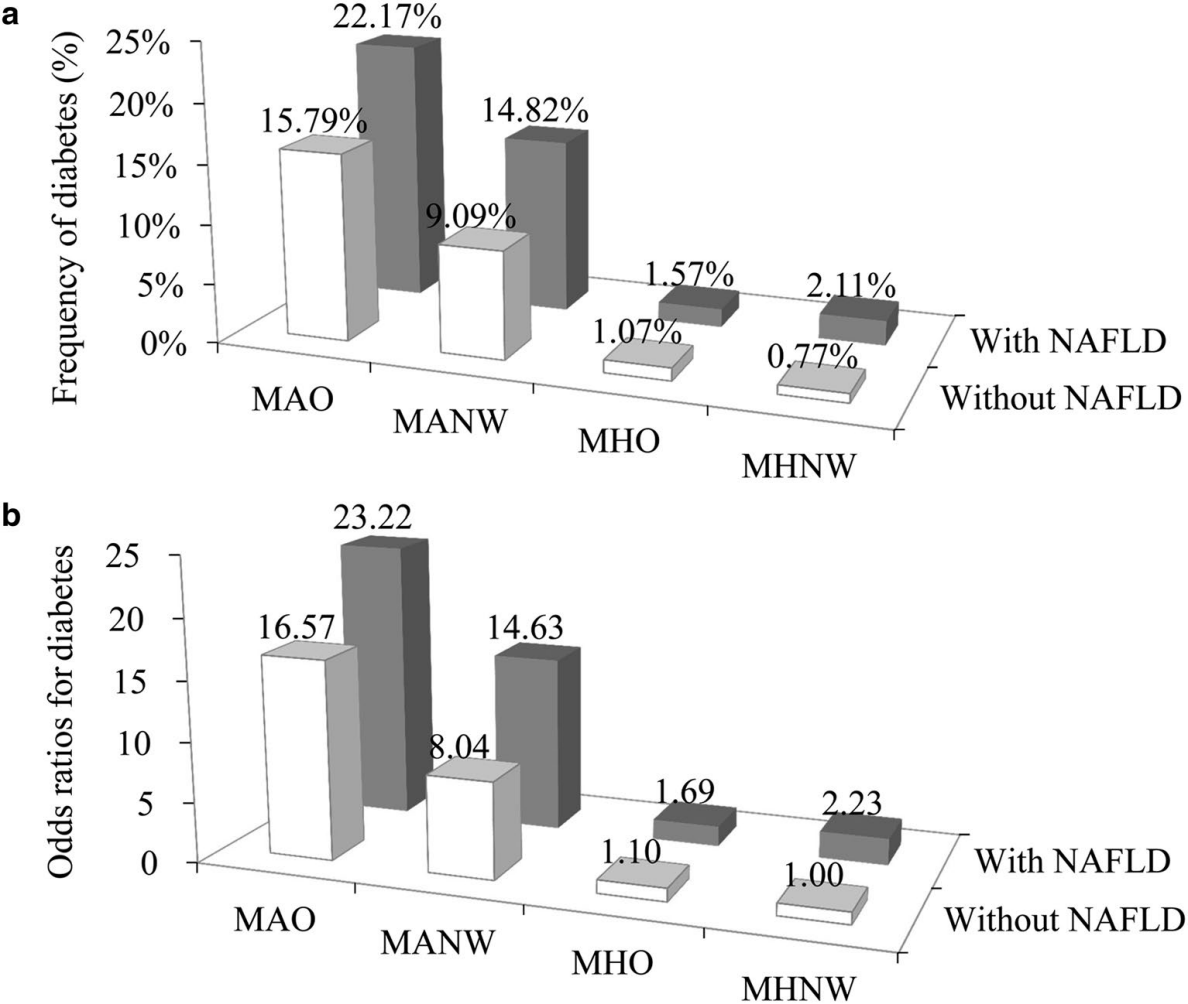
Combined effect of the presence of NAFLD and body size phenotypes on diabetes risk. Combined effect of the presence of NAFLD and body size phenotypes on the frequency of diabetes (**a**); Combined effect of the presence of NAFLD and body size phenotypes on the odds of diabetes (**b**). Odds ratios (95 % confidence intervals) of diabetes for participants categorized by cross-classification of four body size phenotypes and either the presence or absence of NAFLD were adjusted for age, sex, and triglycerides/HDL cholesterol. *MHNW* metabolically healthy normal weight, *MHO* metabolically healthy overweight or obese, *MANW* metabolically at-risk normal weight, and *MAO* metabolically at-risk overweight or obese. Each of the four body size phenotypes was defined in the definition section

We then examined the joint effects of body size phenotypes and the NAFLD status on the diabetes prevalence (Fig. 3a). The MHNW/without NAFLD group (0.77 %) had a similar frequency of diabetes to the MHO/without NAFLD group (1.07 %), while MHNW/with NAFLD had a higher frequency of diabetes (2.11 %). The frequency of diabetes in the MHO/without NAFLD group (1.07 %) was a little lower than that in the MHO/with NAFLD group (1.57 %). The frequency of diabetes was substantially higher in the MAO/without NAFLD group (15.79 %).

Multivariable-adjusted ORs for diabetes associated with overweight/obesity, metabolically at-risk, NAFLD, and their joint effects were displayed in Fig. 3b. Overweight/obesity was not associated with diabetes risk (1.1 [0.61–1.98]), whereas both metabolically at-risk and NAFLD increased the multivariable ORs for diabetes. Compared to the lowest risk group (the MHNW/without NAFLD group), the MHNW/with NAFLD group, the MHO/without NAFLD group, the MHO/with NAFLD group, the MANW/without NAFLD group, the MANW/with NAFLD group, the MAO/without NAFLD group, and the MAO/with NAFLD group had an multivariate-adjusted OR of 2.23 (95 % CI 1.05–5.14), 1.10 (95 % CI 0.61–1.98), 1.69 (95 % CI 1.01–2.53), 8.04 (95 % CI 5.0–12.09), 14.63 (95 % CI 9.77–21.91), 16.57 (95 % CI 10.47–26.24), and 23.22 (95 % CI 13.96–38.63) for diabetes risk, respectively. The trend for increasing ORs was more prominent among subjects with NAFLD (P for the interaction term body size phenotypes *NAFLD status <0.001).

## Discussion

In the present study, we have described for the first time the clinical characteristics of the subjects stratified by body size phenotypes and NAFLD status. We found that NAFLD had a stronger association than overweight/obesity while a weaker association than metabolically at-risk with unfavorable lipid and glucose profiles. We also found that, for a given metabolic status, the associations of NAFLD with unfavorable lipid and glucose profiles were more pronounced in normal-weight subjects than in overweight/obese subjects, emphasizing the importance of body fat distribution in the involvement of diabetes. In addition, we illustrated for the first time how the three factors (NAFLD, overweight/obesity, and metabolic at-risk) cluster together in both the whole population and the diabetic population. We found a significant dissociation between these three factors in the whole population, with only 27.1 % subjects with the presence of all the three factors occurring together. The simultaneous presence of the three factors was common (56.16 %) in diabetic population, suggesting the frequency and importance of the clustering of these three factors for diabetes. Furthermore, we have examined the combined and separate associations of NAFLD, overweight/obesity, and metabolically at-risk with diabetes risk. We found that NAFLD and metabolically at-risk both independently associated with diabetes risk, with stronger associations seen for metabolically at-risk. We also found that the occurrence of all three factors together had the strongest association with diabetes.

Accumulating evidence shows that NAFLD is strongly associated with CVD risk factors [25, 26]. However, most of the studies regarding the association of NAFLD with CVD risk factors focused on obese subjects. Few studies have examined whether the diabetes risk associated with NAFLD is influenced by BMI categories or by metabolic status. A recent study that examined the associations of NAFLD with CVD risk factors according to BMI categories reported a more pronounced association in non-obese group than in obese group [8], indicating that the associations of NAFLD with CVD risk factors may vary by a patient’s BMI status. The present analysis extended these findings to report the association between NAFLD and diabetes risk according to body size phenotypes (that is, cross-classification of BMI categories and metabolic status). We found that, in the absence of metabolically at-risk, NAFLD was associated with increased diabetes risk, regardless of obesity status. However, the association was more pronounced in non-obese group than in overweight/obese group. In the presence of metabolically at-risk, the significant association between NAFLD and diabetes was lost. Taken together, the association between NAFLD and diabetes varies both by a patient’s BMI category and metabolic status. Although explanations for the results remain to be elucidated, it is probably related to differences in regional adipose tissue distribution [27]. BMI provides a general measure of obesity but cannot discriminate among visceral, ectopic, and subcutaneous fat accumulation. It is possible that visceral fat accumulation or ectopic fat deposition in tissues such as liver and pancreas have a stronger relationship with obesity-associated comorbidities than BMI [27–29]. The anatomic, cellular and molecular features of visceral or ectopic fat define the obesity-associated outcomes [27]. The significance of other factors such as the generational transfer of metabolic phenotype or NAFLD to offspring which occurred partly through epigenetic changes or micro-biota [30], and sleep apnea [31] may also explain our results. Obstructive sleep apnea, which often coexists with obesity, can mediate fatty pancreas through tissue hypoxia and lipolysis [31]. Evidence shows that fatty pancreas is a sensitive marker for NAFLD [32], and metabolic syndrome [29].

The concomitant presence of overweight/obesity, NAFLD, and metabolically at-risk is common [33, 34]. However, in the present study, we observed considerable dissociation between the three risk factors, which might explain the different macrovascular complication phenotypes in diabetic patients [35]. According to the adipocyte expandability hypothesis, the subcutaneous fat acts as a metabolic sink, buffering dietary fat to limit their deposition in other organs [36–38]. When increased energy intake exceeded the subcutaneous adipose tissue’s capacity for buffering, lipid will be ectopically deposited in ectopic sites such as liver and the visceral component of abdominal fat. The adipocyte overflow hypothesis infers that some obese individuals, referred as metabolically healthy obesity, are characterized by increased lipid deposition in subcutaneous depot, and thus are protected against obesity-related metabolic disturbances. Conversely, for some obese individuals, called metabolically at-risk obesity, subcutaneous adipose tissue may reach its maximal storage capacity, causing an overflow of fatty acids into ectopic sites, inducing lipotoxicities that in turn lead to diabetes. The remarkable variation in metabolic status for any given BMI observed in a study [39] and the remarkable variation in metabolic status and NAFLD status for any given BMI category observed in our present study further supported the notion.

The dissociation between the three factors results in different combinations of the three factors, conferring differential odds of diabetes and different macrovascular complication phenotypes in diabetic patients [35], as each of the three factors act via different pathogenetic mechanisms to increase diabetes risk. It is well known that increased BMI is associated with pro-inflammatory molecules and atherogenic lipid biomarkers, which are capable of inhibiting insulin signaling. NAFLD increases diabetes risk via mechanisms that increase the secretion of hepatokines such as fetuin-A, and plasminogen-activator inhibitor-1 [40], increase gluconeogenesis, decrease glycogen synthesis, and inhibit insulin signaling [41, 42]; Metabolically at-risk is closely correlated with visceral adiposity [43]. Visceral adiposity, having a greater endocrine activity than does subcutaneous fat [27], may affect risk of diabetes via an effect on the secretion of pro-inflammatory molecules capable of inducing insulin resistance, increased rates of lipolysis that induce excessive free fatty acid and resistine release and thus leading to lipotoxicity and insulin resistance.

Although we observed greater ORs for diabetes associated with metabolically at-risk compared with NAFLD, being NAFLD was associated with poorer lipid and glucose profiles within the same metabolic status. Among both the normal-weight group and overweight/obese group, the most favorable lipid and glucose profiles were found in subjects with metabolically health/without NAFLD. In addition, there was a marked increase in odds of diabetes in subjects with metabolically at-risk/with NAFLD, regardless of obesity status. The fact that metabolically at-risk and NAFLD have independent effects on each other and have synergistic effects on diabetes risk suggests that targeted specific approaches to preventing visceral fat or ectopic fat accumulation or to reversing the visceral fat or ectopic fat accumulation may have a considerable impact on decreasing diabetes risk. The presence of all the three factors occurring together, which was the most frequent combination of the three factors among diabetic subjects, had the strongest association with diabetes, indicating that an optimal prevention strategy for diabetes should emphasize amelioration of the effects of each of the three factors.

Several prospective cohort studies suggest that MHO phenotype might be associated with a nonsignificant or significant increased risk of diabetes incidence [33, 44–47]. Until now, there is no consensus on how to define the MHO phenotype. The absence of a universal definition for MHO phenotype might result in substantial misclassification of individuals who actually have a high-risk phenotype as having a low-risk phenotype. Our results that MHO without NAFLD group did not have a significantly increased OR for diabetes and that MHO with NAFLD group had an OR of 1.69 (95 % CI 1.01–2.53) for diabetes suggest that NAFLD could play important roles in differentiating obese phenotypes at high risk of diabetes. In the present study, 31.72 % MHO individuals suffered from NAFLD. Hence, adding information on the presence of NAFLD into the assessment of MHO phenotype would contribute to assess whether an individual was actually in a metabolically healthy state. In addition, in the obesity state, metabolic at-risk favors the storage of fat in the liver and heralds a higher risk of nonalcoholic steatohepatitis and advanced fibrosis [48]. Nonalcoholic steatohepatitis forecasts an increased risk of CVD mortality [49]. Hence, exploring how the three factors (NAFLD, overweight/obesity, and metabolic at-risk) cluster together could help to early identification of patients with nonalcoholic steatohepatitis and might have clinical implications for developing diagnostic and therapeutic strategies targeting CVD.

There are several limitations. The cross-sectional design implies that no causal relationship can be drawn. We only had data on Chinese adults comprised of selected populations (industrial employees and retired workers) with a preponderance of men, thus, extrapolating results to the Chinese general population or to other racial or ethnic population should be interpreted cautiously. Since NAFLD was diagnosed by ultrasonography, which is a reasonably accurate technique for detecting modest amounts of liver fat (>30 % liver fat infiltration), participants with minor amounts of fatty infiltration might not have been captured. That NAFLD was not confirmed by liver biopsy was also a limitation. A standard 75-g oral glucose tolerance test was not performed, which might suggest an underestimation of the diabetes prevalence.

## Conclusions

The association of NAFLD with diabetes is more pronounced in normal-weight individuals than in overweight/obese individuals among participants without metabolic at-risk. There is a significant dissociation of the three factors (overweight/obesity, NAFLD, and metabolically at-risk) in the whole population, while the three factors commonly occur together in diabetic individuals. Of the three factors, overweight/obesity had the weakest association with diabetes and metabolically at-risk had the strongest association. The presence of all three risk factors occurring together strikingly and markedly increases the diabetes risk. NAFLD should be considered as a more meaningful predictor of diabetes in MHO individuals.

## Abbreviations

NAFLD: nonalcoholic fatty liver disease
BMI: body mass index
OR: odds ratio
CVD: cardiovascular disease
WHO: World Health Organization
BP: blood pressure
FBG: fasting blood glucose
TC: total cholesterol
TG: triglycerides
LDL-C: low-density lipoprotein cholesterol
HDL-C: high-density lipoprotein cholesterol
ALT: alanine aminotransferase
MHNW: metabolically healthy normal weight
MANW: metabolically abnormal normal weight
MHOW: metabolically healthy overweight or obese
MAO: metabolically abnormal overweight or obese
SE: standard error
CI: confidence interval
